# The willingness of private-sector doctors to manage public-sector HIV/AIDS patients in the eThekwini metropolitan region of KwaZulu-Natal

**DOI:** 10.4102/phcfm.v2i1.102

**Published:** 2010-03-24

**Authors:** Panjasaram Naidoo, Champaklal C. Jinabhai, Myra Taylor

**Affiliations:** 1 School of Pharmacy and Pharmacology, University of KwaZulu-Natal, Westville campus, South Africa; 2 Department of Public Health Medicine, University of KwaZulu-Natal, Howard College campus, South Africa

**Keywords:** private sector, doctors, public sector, patients, HIV/AIDS

## Abstract

**Background:**

South Africa is severely affected by the AIDS pandemic and this has resulted in an already under-resourced public sector being placed under further stress, while there remains a vibrant private sector. To address some of the resource and personnel shortages facing the public sector in South Africa, partnerships between the public and private sectors are slowly being forged. However, little is known about the willingness of private-sector doctors in the eThekwini Metropolitan (Metro) region of KwaZulu-Natal, South Africa to manage public-sector HIV and AIDS patients.

**Objectives:**

To gauge the willingness of private-sector doctor to manage public-sector HIV and AIDS patients and to describe factors that may influence their responses.

**Method:**

A descriptive cross-sectional study was undertaken among private-sector doctors, both general practitioners (GPs) and specialists, working in the eThekwini Metro, using an anonymous, structured questionnaire to investigate their willingness to manage public-sector HIV and AIDS patients and the factors associated with their responses. Chi-square and independent t-tests were used to evaluate associations. Odds ratios were determined using a binary logistic regression model. A *p* value < 0.05 was considered statistically significant.

**Results:**

Most of the doctors were male GPs aged 30–50 years who had been in practice for more than 10 years. Of these, 133 (77.8%) were willing to manage public-sector HIV and AIDS patients, with 105 (78.9%) reporting adequate knowledge, 99 (74.4%) adequate time, and 83 (62.4%) adequate infrastructure. Of the 38 (22.2%) that were unwilling to manage these patients, more than 80% cited a lack of time, knowledge and infrastructure to manage them. Another reason cited by five doctors (3.8%) who were unwilling, was the distance from public-sector facilities. Of the 33 specialist doctors, 14 (42.4%) indicated that they would not be willing to manage public-sector HIV and AIDS patients, compared with only 24 (17.4%) of the 138 GPs (*p* < 0.01).

**Conclusion:**

Many private-sector doctors are willing to manage public-sector HIV and AIDS patients in the eThekwini Metro, potentially removing some of the current burden on the public health sector.

## INTRODUCTION

Health systems in developing countries are in crisis; the deficits include a lack of sufficient health professionals, inadequate finance and poor quality of service, together with poor infrastructure, which includes a lack of reliable water, sanitation and electricity.^1^ Without adequate infrastructure, doctors and nurses cannot provide quality care even when they are available. Underlying this is a serious shortage of skilled, trained managers and the deficit is greatest in sub-Saharan Africa, where 17% of the total health workforce is employed as managers, compared to 33% globally. Many countries are now engaging the private sector in partnerships with the public sector as a means of rebuilding their infrastructure and improving access to services.^1^

To address some of the resource and personnel shortages facing the public health sector in South Africa, partnerships between the public and private health sectors are slowly being forged. In May 2006, the Minister of Health launched the National Consultative Health Forum^2^ to discuss key strategic health issues, including tuberculosis, HIV and AIDS, recruitment and retention of health professionals, and transformation of the health sector.^3^ South Africa has an extensive antiretroviral treatment programme: 371 731 patients were initiated on antiretroviral treatment in 2007, whilst approximately 76 217 (22%) received treatment funded by medical schemes, private-sector and development partners for 2006, a number which increased to 28% in 2007.^4^ Governments and donors are increasingly considering the private sector as a potential partner in addressing the growing demand for sustainable HIV and AIDS treatment. Given the weaknesses and strengths of both sectors, a partnership between the public and private sectors has became a viable policy option since neither the public nor the private sector alone can deliver competent, accessible and affordable health care.^5^

The AIDS pandemic has severely affected South Africa, with an estimated 5.7 million South Africans living with HIV and AIDS in 2007.^6^ HIV and AIDS thus constitutes the major part of the South African burden of disease.^7^ High levels of internalised stigma encourage people to hide their condition^8^ and many HIV and AIDS patients prefer to consult a private-sector doctor in order to avoid the stigma.^9^ However, little is known about willingness of private-sector doctors to manage public-sector HIV and AIDS patients. This study investigated the issue in the eThekwini Metropolitan (Metro) area of KwaZulu-Natal (KZN) and described possible factors that could influence doctors’ responses.

## METHOD

### Study design, study area and sample population

This descriptive cross-sectional study of private general practitioners and specialists was undertaken in the eThekwini Metro of KZN. This province has the largest provincial population in South Africa, with just over 10 million people (20.9% of the total population of 47.9 million),^10^ and is the province with the highest prevalence of HIV, as indicated by antenatal clinic attendees’ data (39.1% in 2006, compared to the national figure of 29.1%).^11^ The eThekwini Metro has a population of 3 090 126, comprising 51.9% (1 605 080) women and 48.1% (1 485 046) men.^12^ Most of the eThekwini Metro is urban (central) and suburban (south, north and west), with a small rural constituency (inner west and further south).

### Study sample

The sample population comprised all general practitioners (GPs) and specialists that work in the private health care sector of the eThekwini Metro area. These doctors are remunerated either by patients paying cash or via a medical aid scheme. They are independent of any funding from the government. A comprehensive list of 1 255 GPs and specialists practicing in the eThekwini Metro was obtained from the Medpages Directory, the KwaZulu-Natal Managed Care Coalition (KZNMCC, a private doctor grouping), the private doctors’ guilds, the Lancet Clinic Courier database, and the Southern African HIV Clinicians’ Society. This was done to ensure that all eThekwini medical practitioners in the private sector were included. The study initially identified doctors that managed HIV and AIDS patients in the private sector, and/or their reasons for not doing so, and their training needs (Phase 1).^13^ There were 235 doctors from the Phase 1 study who indicated that they managed HIV and AIDS patients, only 190 of whom indicated their willingness to participate in Phase 2 of the study. The second phase was undertaken to determine the doctors’ willingness to manage public-sector patients in the eThekwini Metro, as well as to investigate the motivations for their choice.

Trained field workers were allocated to the doctors who had indicated their willingness to participate in this study. The doctors were first telephoned, to ensure their availability and to confirm their consent. Most of the questionnaires were hand delivered and later collected. A few questionnaires were faxed to participants and a few were faxed back by the doctors.

No records of the doctors’ name or contact details were kept. The data were captured and analysed using SPSS version 15. Factors influencing doctors’ willingness to treat patients, such as time, knowledge and infrastructure, were ranked from 0 to 3. The chi-square statistic was used for categorical data analysis and the independent samples t-test for continuous data. A binary logistical regression model, with all four factors entered as independent variables, was applied using a backward stepwise fitting method. A *p* value < 0.05 was considered statistically significant.

Ethical approval for the study was obtained from the Ethics Committee of the Nelson R. Mandela School of Medicine, University of KwaZulu-Natal.

## RESULTS

The results provided a demographic profile of the doctors (Table 1), their willingness to manage public-sector patients (Table 2), and their reported resources of time, knowledge and infrastructure (Table 3). A response rate of 90% (n = 171) was obtained.

**TABLE 1:** Demographic profile of private-sector doctors in the eThekwini Metro

Variable	Number (%)
Sex*	
Male	138 (85.7)
Female	23 (14.3)
**Age (years)†**	
30–40	38 (23.9)
41–50	70 (44)
51–60	33 (20.8)
61–70	15 (9.4)
> 70	3 (1.9)
**Speciality‡**	
GP	138 (80.7)
Specialist	33 (19.3)
**Length of practice (years)§**	
1–10	41 (24.4)
11–20	66 (39.3)
21–30	41 (24.4)
31–40	17 (10.1)
> 40	3 (1.8)
**Area of practice¶**	
Central eThekwini	56 (33.3)
South eThekwini	52 (31)
North eThekwini	32 (19)
West eThekwini	10 (6)
Other (working in more than one area)	18 (10.7)

* n = 161

† n = 159

‡ n = 171

§ n = 168

¶ n = 168

**TABLE 2:** Doctors’ willingness to manage public-sector HIV and AIDS patients

Reasons influencing doctors’ decisions						

	Willing: 133 (77.8%)			Unwilling: 38 (22.2%)*		
				
Factors	Yes	No		Yes	No	*P* value
Adequate time	99 (74.4%)	34 (25.6%)		1 (2.7%)	36 (97.3%)	0
Adequate knowledge	105 (78.9%)	28 (21.1%)		5 (13.5%)	32 (86.5%)	0
Adequate infrastructure	83 (62.4%)	50 (37.6%)		3 (8.1%)	34 (91.9%)	0

* While 38 respondents indicated they were not willing to manage public-sector HIV/AIDS patients, only 37 provided a reason for their decision.

**TABLE 3:** Association between doctors’ willingness to treat public-sector HIV and AIDS patients and the availability of resources (time, knowledge, infrastructure)

Doctors’ willingness to manage HIV/AIDS patients	Total availability of resources				
	0*	**1†**	**2‡**	**3§**	**Total**
					
Yes	6 (4.5%)	29 (21.8%)	36 (27.1%)	62 (46.6%)	133
No	31 (81.6%)	4 (10.5%)	3 (7.9%)	0	38

Pearsons’ chi-square value is 105.555, *p* value < 0.01.

* Lack of time, knowledge and infrastructure.

† Availability of one resource, either adequate time, or knowledge or infrastructure.

‡ Availability of two resources, either adequate time, or knowledge or infrastructure.

§ Availability of all three resources: adequate time, knowledge and infrastructure.

### Demographic profile of private-sector doctors

Over 80% of the respondents were men and the majority of the doctors were GPs between the ages of 30 and 50 years. Three-quarters of the doctors had been in practice for more than 10 years and were based in the central and southern areas of the eThekwini Metro (Table 1). The number of doctors that were willing to manage public-sector HIV-infected patients and reasons that influenced their decisions are depicted in Table 2.

Of the 77.8% (n = 133) of doctors who were willing to manage public-sector HIV and AIDS patients, the majority indicated that they had adequate time and knowledge, but fewer considered that they had adequate infrastructure. However, some doctors who were willing to manage these patients still acknowledged that they did not have adequate time, knowledge or infrastructure. There were significant differences amongst the doctors willing/unwilling to manage public-sector patients: lack of time, knowledge and infrastructure were reported by over 85% of the unwilling doctors (*p* < 0.005). However, they comprised less than a quarter of the sample. Another reason cited by five doctors (3.8%) was the distance from public-sector facilities. The demographic variables such as age, sex, number of years in practice and area of practice were not associated with doctors’ willingness to manage public-sector HIV and AIDS patients.

There were, however, significant differences between specialists and GPs in their willingness to manage public-sector patients. Of the 33 specialists that responded, 14 (42.4%) indicated that they would not be willing to manage public-sector patients, whilst only 24 (17.4%) of the 138 GPs indicated their unwillingness to manage (*p* < 0.01). The relationship between the availability of resources and the doctors’ willingness to manage public-sector HIV and AIDS patients is ranked and shown in Table 3.

As can be seen in Table 3, 81.6% of the doctors who were not willing to manage such patients indicated that they did not have adequate time, knowledge or infrastructure to be able to manage these patients. The significant association between the ranking of the three factors (time, knowledge and infrastructure) suggests that the availability of resources influenced the doctors’ decision whether or not to manage public-sector HIV and AIDS patients (*p* < 0.01).

Table 4 presents a model of doctors willing to manage public-sector HIV and AIDS patients. A statistical model ranking all the factors showed that time was the most important predictor, followed by knowledge. Distance from public-sector facilities was also significant but not as important as the other two factors. Infrastructure became non-significant once the other factors were adjusted for. However, the confidence intervals were very wide, suggesting that the model should be interpreted with caution. This may be due to the small size of the sample.

**TABLE 4:** Model of doctors willing to manage public-sector HIV and AIDS patients

Factors	B (SE)	Significance	OR	95% CI for OR	
				**Lower**	**Upper**
					
Time	4.88 (1.11)	0	131.55	14.86	1164.75
Knowledge	3.39 (0.67)	0	29.646	7.99	110.01
Distance from public-sector facilities	1.90 (0.96)	0.047	6.67	1.03	43.42

Variables entered were time, knowledge, distance from public-sector facilities and infrastructure. B, Beta (logistic regression coefficient). SE, Standard error of coefficient. OR, Odds ratio.

### Counselling of HIV and AIDS patients

Of the 159 doctors who responded about counselling of HIV and AIDS patients, only 67 (42.1%) indicated that they had sufficient time to counsel these patients.

### Remuneration

When asked whether they were adequately remunerated by medical aid schemes, only 14 (20.9%) of the 67 doctors perceived that they were adequately remunerated. Although 103 (77.4%) felt that they were inadequately remunerated by medical aids, they were nonetheless prepared to manage public-sector HIV and AIDS patients.

## DISCUSSION

The results suggest that many private-sector doctors who manage HIV and AIDS patients are also willing to manage public-sector HIV and AIDS patients. It was noted in this study that the GPs were more willing than the specialists to manage public-sector patients. This could be because there are fewer specialists, particularly in the field of HIV and AIDS in the eThekwini Metro; specialists do have GPs referring their HIV and AIDS patients to them.^13^ Normally when patients are referred they are at a more advanced stage than patients seen by GPs. Specialists may have encountered failure in the management of these patients, which may have resulted in a lack of confidence in their ability to manage HIV-infected patients.^13^ In addition, specialists treat many other infectious diseases and may not regard themselves as HIV experts.^14^

The factors that were associated with the willingness of doctors to manage public-sector HIV and AIDS patients, such as time, knowledge and infrastructure, would need to be improved as they may have contributed to doctors’ unwillingness to manage these patients. Although the majority of the respondents were willing to treat public-sector HIV and AIDS patients, only 46% were confident that they had all three of the components, that is, adequate time, knowledge and infrastructure, to manage these patients. Several past studies that have examined the role of doctors in the management of HIV and AIDS patients have shown that doctors lacked the necessary knowledge and competency required for the management of AIDS.^15^ In the 1990s, many studies recognised that the lack of, or inadequate, medical/clinical knowledge to treat HIV and AIDS was a barrier that resulted in doctors not wanting to manage these patients.^16^ The present study confirms these trends, because lack of knowledge was associated with doctors not wanting to manage public-sector HIV and AIDS patients. Even though these doctors were managing private-sector patients, they may require additional knowledge to manage an increased load of patients, as would be the case if they were to manage both private- and public-sector HIV and AIDS patients. Other studies have found that doctors with lower levels of knowledge saw fewer patients^17^ and that doctors who had high patient volumes tended to be better informed.^14^

Coupled to inadequate HIV training or knowledge was the time factor; as a result of a lack of training and experience in managing HIV and AIDS patients, doctors may need to spend more time managing these patients. Such doctors could then manage fewer patients compared to the number that could be managed by their more experienced colleagues, resulting in more time subsequently being spent on fewer patients.^13^ Lack of time or a demand on the doctors’ time was a commonly cited reason for doctors not willing to manage HIV and AIDS patients in the developed world.^16^ Our findings are consistent with other studies, where lack of infrastructure, or poor infrastructure, such as lack of support staff, the structure of a general practice, lack of specialty backup support for patients who develop complications, or lack of community social services or resources,^16^ have presented barriers to doctors willing to manage HIV and AIDS patients.

Private-sector doctors are important in the delivery of health care. An recent Indian study found that although there was an abundance of HIV testing in the private sector, it was accompanied by inappropriate practices and inadequate knowledge, reflecting deficiencies in the implementation of policy guidelines.^18^ Despite this, the needs of private providers, who are a major source of health care in India, have to be acknowledged and, with the use of supportive and regulatory mechanisms, this sector could be used effectively to provide better HIV testing services.^18^ This sentiment is echoed by the CEO of the KZNMCC, who stated that

*private–public partnerships require constructive engagement between the two sectors, whereby the resources, expertise and personnel within the private sector can be harnessed to create a solution that achieves the objective of health for all. Achieving this requires that both sectors be regulated optimally and relevantly, and that this partnership is the solution to ensure that there is a sustainable health system five years hence*.^19^

Public–private partnerships (PPP) can take various forms, depending on whether the private sector is involved in financing and/or providing health care. Two of the many categories could be, (1) where the public sector pays the private sector for the caring of public-sector patients, for example HIV and AIDS patients can be diverted from hospitals to accredited private health care providers and paid for per capita and (2) where the private-sector doctor works for a specified number of hours in a public-sector facility.^20^

However, there are many challenges that face PPP, including the pervasive mistrust between the sectors, which need to be overcome. There should also be strong governmental leadership and political will to form this partnership, with all partners being well-informed about the business plan as well as about one other. There should be coordination between the Ministries of Health and Finance, the latter ultimately approving the partnership agreement, with the Ministry of Health benefiting from the additional health infrastructure and charged with managing the private partner. There should also be support from the communities, because civil society has a significant role to play in health care delivery in sub-Saharan Africa.^1^

National Health Insurance, which insures the national population for the costs of health care, has been advocated as part of a programme of health care reform. It may be administered by the public sector, the private sector, or a combination of both sectors. Funding mechanisms vary with the particular programme and the country. The CEO of KZNMCC has suggested that

*with the proposed national health insurance in Government, there is a great need for both sectors to work together. Private practitioners and private health care generally are resource-rich and can help ensure the seamless implementation of a NHI programme*.^19^

## LIMITATIONS

Firstly, this study considered the basic requirements of GPs and specialists, namely time, knowledge, infrastructure and distance from public-sector facilities, in determining their willingness to manage public-sector HIV and AIDS patients, but there may be additional factors that influence their decisions. Secondly, the results of this study may not be applicable to all private-sector doctors in South Africa, because the sample size was relatively small and confined to the eThekwini Metro. Thirdly, this was a self-reported study; the reliability of self-reporting is difficult to substantiate because information was collected and analysed based on what the doctors reported. Finally, as a cross-sectional study, the direction of the association may not be causal.

## CONCLUSION AND RECOMMENDATIONS

There is a clear willingness in the private sector to help government manage the HIV-infected population in South Africa. It now depends on the government to explore this possibility further and establish a well-regulated partnership with the private sector to share resources in the management of HIV and AIDS patients and to provide a framework of incentives, both financial and non-financial. Various models could be examined in order to ensure an effective partnership and service delivery. Urgent educational interventions should be sought in order to improve the knowledge base of private-sector doctors to HIV and AIDS management. Structured continuing medical education programmes and workshops should be conducted in order to facilitate the broadening of HIV and AIDS management amongst private-sector doctors.
